# The Role of Cations in Resorcinol–Formaldehyde Gel Textural Characteristics

**DOI:** 10.3390/gels8010060

**Published:** 2022-01-15

**Authors:** Stewart J. Taylor, Liu Yang, Ashleigh J. Fletcher

**Affiliations:** 1 Department of Chemical and Process Engineering, University of Strathclyde, Glasgow G1 1XJ, UK; essjaytee88@hotmail.com; 2 Department of Mechanical & Aerospace Engineering, University of Strathclyde, Glasgow G1 1XJ, UK; liu.yang@strath.ac.uk

**Keywords:** resorcinol–formaldehyde, xerogels, catalyst, porous, sol-gel, Hofmeister series

## Abstract

The production of resorcinol–formaldehyde xerogels has yielded insight into the gelation processes underpinning their structures. In this work, the role of the cation species from the catalyst is probed by studying the simultaneous addition of sodium carbonate and calcium carbonate to a resorcinol–formaldehyde mixture. Twenty-eight xerogels were prepared by varying the solids content, catalyst concentration, and catalyst composition, and each was analysed for its textural properties, including the surface area and average pore diameter. The results indicate that the role of the cation is linked to the stabilisation of the clusters formed within the system, and that the Group II catalyst causes the salting out of the oligomers, resulting in fewer, larger clusters, hence, an increase in pore size and a broadening of the pore size distribution. The results provide insight into how these systems can be further controlled to create tailored porous materials for a range of applications.

## 1. Introduction

Resorcinol–formaldehyde (RF) gels are a family of organic, porous materials that have seen widespread study since their discovery by Pekala in 1989 [1]. The gels can be classified into three sub-categories on the basis of the drying process used in their production: aerogels (supercritically dried), xerogels (sub-atmospherically dried), and cryogels (freeze-dried) [2,3,4]. The rapid and comparatively simple synthesis of xerogels has made them the focus of much research, with practicable, scaled synthesis underpinning their potential applications (as a consequence of their porous nature) in a range of sectors, including catalyst supports [5], gas storage systems [6], soil remediation [7], fuel cells production [8], and insulation [9]. All of these aforementioned applications require a porous material in which the internal structure, in terms of pore size and connectivity, can be carefully controlled. As such, porous materials can be optimised for a particular application, and RF aerogels possess the ability to exhibit varying porous textures, dependent on the synthesis conditions chosen.

**Figure 1:**
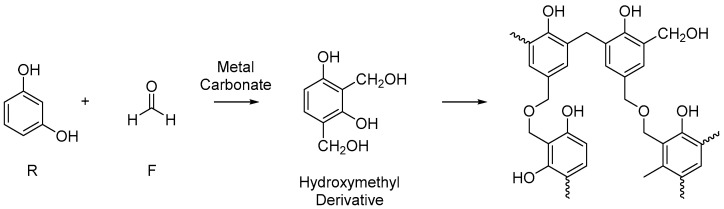
General reaction scheme in the formation of resorcinol–formaldehyde gel.

The generally accepted reaction scheme in the sol-gel polycondensation of resorcinol (R) and formaldehyde (F) is given in Figure 1, with the reaction typically carried out at elevated temperatures. An initial addition reaction between R and F forms the hydroxymethyl derivative product. This is followed by the condensation of these derivatives into growing oligomeric chains which form clusters, and subsequently, a cross-linked 3D gel network. The metal carbonate of choice is typically sodium carbonate (Na_2_CO_3_), as described by Pekala in his original synthesis, and used comprehensively since. The carbonate acts as a base and promotes the initial reaction between resorcinol and formaldehyde. However, the polycondensation reaction is highly flexible, and a number of synthesis parameters can be varied in order to modify the porous texture of the final aerogel product [2,10]. Commonly, these variables include the composition of the base (both cation, e.g., calcium, and anion, e.g., hydroxide) [11,12,13], the resorcinol-to-carbonate molar ratio (R/C), and the quantity of solids dissolved within a fixed volume of solvent (deionised water) [14]. However, recent work has also shown that both the time allowed for the reaction mixture to be stirred before heating [15] and the shape of the mould used to form the RF aerogel [16] can also have a significant effect on the internal structure of the gel product.

Despite significant levels of investigation into RF xerogels, the complex nature of the competing chemical (e.g., polymerisation) and physical processes (e.g., aggregation) involved in the formation of these materials results in a lack of complete understanding of the gel network development process. However, detailed examination of the formation mechanism through light scattering techniques has resulted in the creation of a cluster growth model; for a given set of reaction variables, the size of the growing clusters is controlled thermodynamically, whereas the number concentration of the clusters is determined kinetically [17]. Furthermore, the metal cation of the basic compound plays a fundamental role in the formation of the clusters and the subsequent gel network by stabilising the colloidal suspension formed by the growing clusters dispersed within the solvent matrix [18]. Work by Taylor et al. [17,18] has shown that ions from a catalytic species within RF syntheses can similarly ‘salt in’ or ‘salt out’ macromolecules from the solution. Recent work has shown that, as RF xerogels are hydrophilic in nature, the ion-surface interactions of RF xerogels is reversed in comparison with the Hofmeister series [19,20], which was originally proposed to order ions according to their ability to stabilise proteins in a solution.

The aim of this current work was to further elucidate the role of the metal cation in the gel formation process. While previous work has shown the effect of the disruptive addition of a second species within the synthetic process [18], this work seeks to determine the effect of the simultaneous addition of competing catalytic bases. Thus, a number of xerogels were produced using Na_2_CO_3_, to provide a singly charged cation, and calcium carbonate (CaCO_3_), to provide a doubly charged cation, at varying R/C molar ratios and solids contents. Furthermore, additional series of xerogels were produced using various mixtures of the two cations in order to monitor any interactions between the cations, and the resultant impact on the final properties of the aerogel products. Low-temperature nitrogen sorption measurements and mercury intrusion porosimetry (MIP) were used to characterise the textural porous properties of the synthesised xerogels, allowing changes in the internal structure of the xerogel to be monitored and quantified.

## 2. Results and Discussion

Two series of RF xerogels were prepared according to the above-described procedure, with each series having a fixed solids content of 20 and 30% weight by volume (hereafter referred to as 20% *w/v* and 30% *w/v*), respectively. In turn, each of these solid-content series could be further categorised into three subsets, as defined by the R/C molar ratio chosen, these being R/C 100, 400, and 600. Each of the subsets comprised five different xerogels differentiated by the respective quantities of each of the metal carbonates used. Table 1 outlines the five different carbonate combinations used.

The stated synthetic parameter combinations produced 30 distinct xerogels; however, when using 20% *w/v*, R/C 600 and a catalyst mixture of either 100% Ca or 75% Ca/25% Na, the system failed to produce a viable gel after three days of gelation and curing at 85 ± 5 °C, which can be ascribed to the combined effects of decreased catalyst concentration and the propensity of the Group II catalysts to form larger clusters, based on particle size [18], thereby reducing crosslinking between the clusters and negatively impacting gel formation. Hence, these xerogels were subsequently excluded from further characterisation tests, and the dataset presented here represents the 28 viable xerogels produced in this work. 

**Figure 2:**
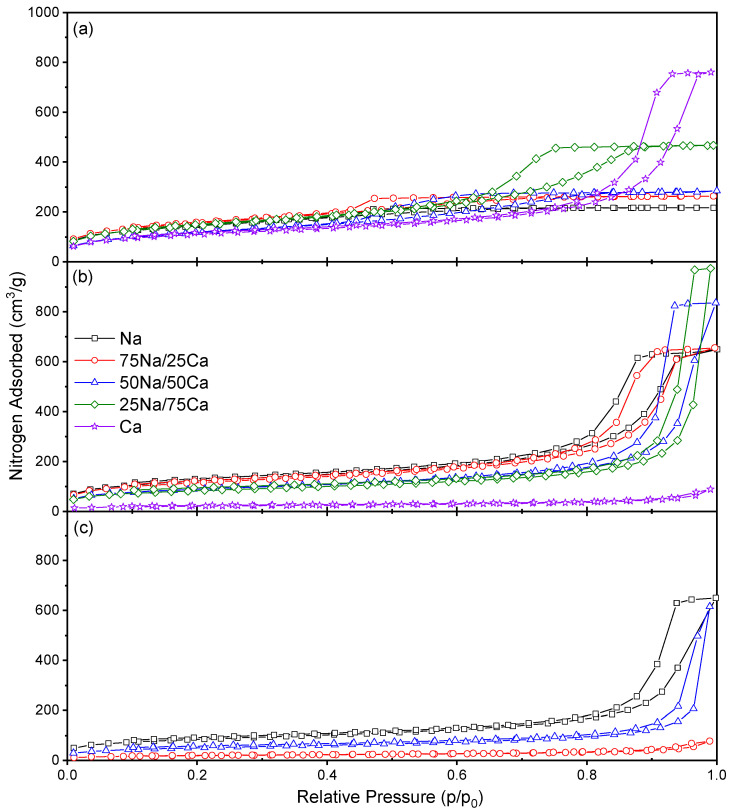
The sorption isotherms obtained for the resorcinol–formaldehyde xerogels produced using 20% solids content and (**a**) R/C 100, (**b**) R/C 400, and (**c**) R/C 600.

**Figure 3:**
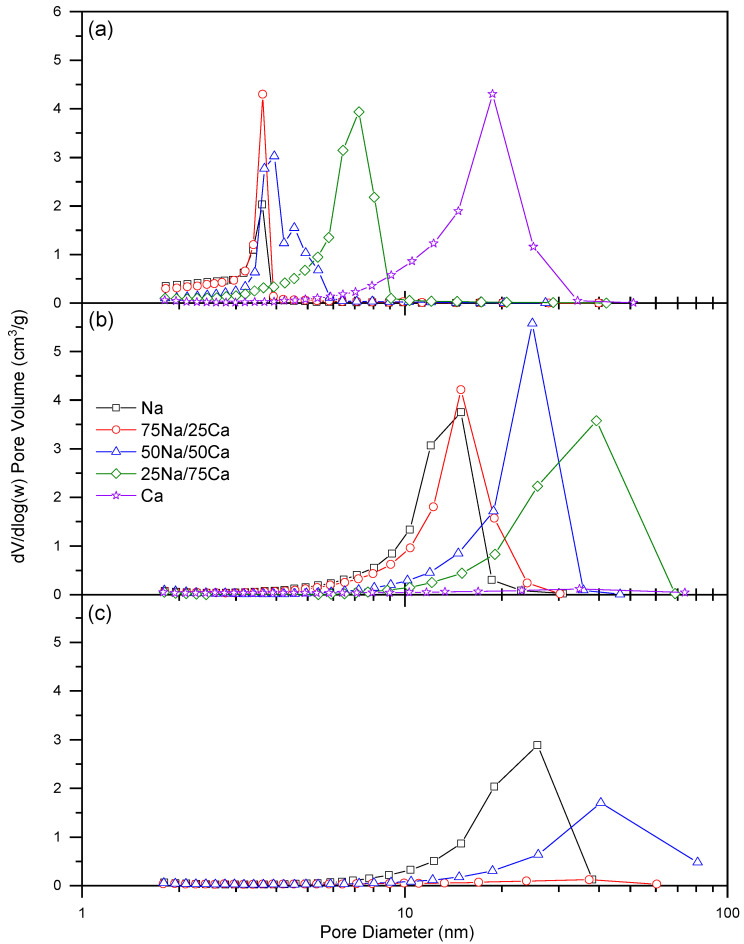
The pore size distributions obtained for the resorcinol–formaldehyde xerogels produced using a 20% solids content and (**a**) R/C 100, (**b**) R/C 400, and (**c**) R/C 600.

**Figure 4:**
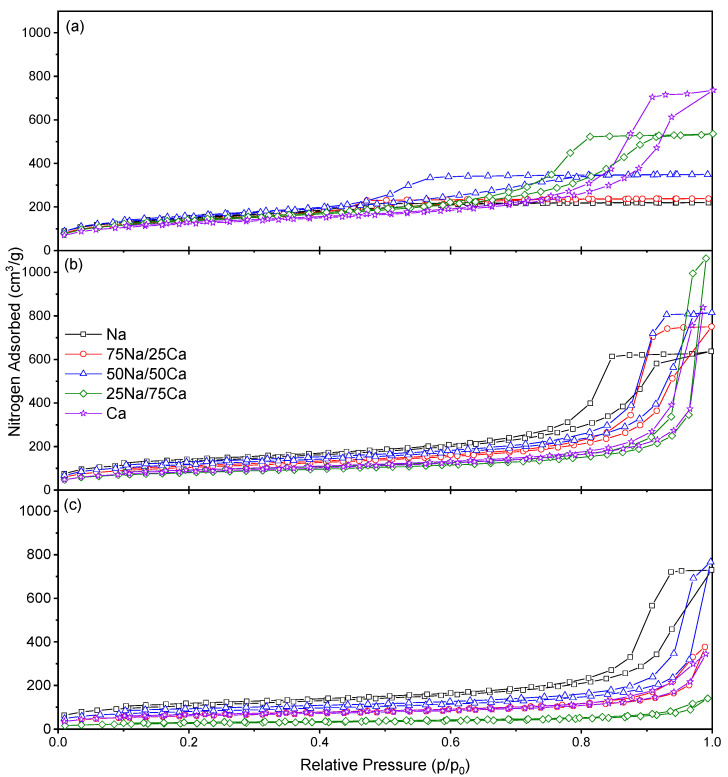
The sorption isotherms obtained for the resorcinol–formaldehyde xerogels produced using a 30% solids content and (**a**) R/C 100, (**b**) R/C 400, and (**c**) R/C 600.

**Figure 5:**
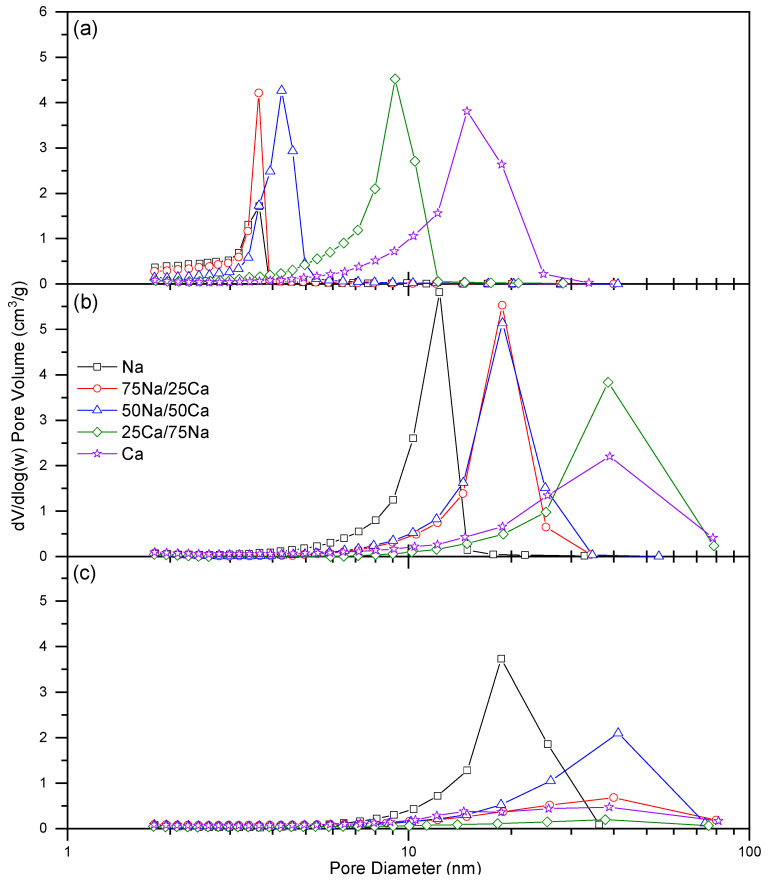
The pore size distributions obtained for the resorcinol–formaldehyde xerogels produced using 30% solids content and (**a**) R/C 100, (**b**) R/C 400, and (**c**) R/C 600.

The adsorption isotherms obtained from the nitrogen sorption measurements are shown in Figure 2 for 20% *w/v* and in Figure 4 for 30% *w/v*, while the associated pore size distributions are shown in Figure 3 for 20% *w/v* and Figure 5 for 30% *w/v*. It is evident that, for the lower solids content, isotherms obtained at all R/C ratios can all be classified as Type IV [21]; however, the closure point for the Type H2(b) hysteresis loops [21] shifts as the Na:Ca ratio is changed. In all cases, the closure point moves towards higher relative pressures as %Ca increases, while the nitrogen uptake generally increases with increasing R/C, as expected from previously reported results [17]. Table 2 shows the data obtained from the analysis of nitrogen sorption isotherms measured for all samples. There is a clear decrease in surface area, with a general increase in pore volume and average pore diameter, moving from the Group I catalyst (Na_2_CO_3_) to the Group II species (CaCO_3_). These same changes are also evident for increasing R/C ratio; decreased catalyst concentration (i.e., increased R/C ratio) has previously been shown to decrease surface area, as a result of the smaller number of nucleation sites creating fewer, larger particles resulting in larger spaces between the particles, hence, a decrease in the available surface area, while increasing the average pore diameter and total pore volume for both Na_2_CO_3_ [17,18] and CaCO_3_ [18]. This latter work presented results for low R/C ratios using CaCO_3_ as a catalyst, i.e., R/C 100 and 200 [18]. The results presented here (Table 2) show an increase in surface area, pore volume, and pore diameters for R/C 100 due to the improved solvent exchange method used, which reduces capillary forces during drying by enhanced the replacement of the entrained solvent. As stated above, the pure CaCO_3_ material produced at a higher R/C failed to produce a viable gel as a result of particle size effects, hence, the trends for this catalyst are inferred from the results for R/C 100 and 400; however, previous results also support these observations [18].

Changes in the closure point of the hysteresis loop can be ascribed to the experimental technique itself used for the Na-rich xerogels, while effects from the solid sorbent are influential for the higher calcium loadings. This is due to the fact that the closure point of a hysteresis loop occurs at a given relative pressure and temperature for a known adsorptive; for nitrogen, this point occurs at p/p^o^ ~0.42 [22] at −196 °C. The closure point moves toward higher p/p^o^ as the calcium content increases; hence, the observed behaviour becomes more characteristic of the material than the adsorptive. Type H2 hysteresis loops are indicative of ‘ink-bottle’ shaped pores and are evidence of differences in condensation and evaporation mechanisms. Hence, the porous structure of the material is likely similar in shape, but the dimensions of the pores are affected. Figure 3 shows the progressive widening of the pore size distributions across all three R/C ratios. It is also notable from Figure 3, and Table 2, that the average pore diameter increases as %Ca increases; this is expected, as the amount of influence from the Group II metal carbonate will increase with concentration. 

Nitrogen isotherms obtained for xerogels synthesised using 30% *w/v* (Figure 4) again exhibit Type IV isotherms with Type H2(b) hysteresis loops [21], indicative of ‘ink-bottle’ pores that again widen as the R/C ratio and %Ca are increased (Table 3). Pore size distributions for the same xerogels (Figure 5) show a similar trend to those obtained for 20% *w/v* with a widening of the pore diameter across the same parameters, and notably, pore dimensions are similar irrespective of the solids content for the same ratio of the catalyst present and the R/C ratio (Table 3). The only differences are evident for 100% Ca; this may be due to the mechanism of gel formation with Group II cations, with these xerogels also showing a trend from porous to non-porous solids where, as stated above, a further reduction in the catalyst results in failure of the gelation process.

Comparison of xerogels obtained using either 100% Na or 100% Ca show that the differences observed for isotherms and pore size distributions are more marked at low R/C ratios, and such behaviour was observed in previous research [18]. Colloidal destabilisation due to a combination of a low solids content and the additional effects of Group II metal ions underpins the inability of Ca_600 20% and 75Ca/25Na_600 20% xerogels to form; this is similar to results often obtained for xerogels synthesised without metal cations (e.g., (NH_4_)_2_CO_3_). Here, the low concentration of Ca^2+^ is a significant contributory factor in the failure to form a viable gel. This is not a factor for the Na^+^ catalysed xerogels, as these tend to consist of closer-packed materials with narrower pore size distributions centred around a smaller pore diameter; hence, there is stabilisation from the closer proximity of the smaller clusters formed. 

The larger pores and pore volumes obtained for the high R/C and 100% Ca xerogels support this theory, and the widening of the pore size distribution for both higher R/C and increased %Ca is also supported by the MIP results, which show larger volumes of macropores for these systems. Our previous work showed that, for the simultaneous addition of two different catalysts, cluster growth was more representative of the pure Group I catalyst at R/C200 [18], indicating that this catalyst was more dominant in these systems; however, such additions were limited to equal molar ratios, and the results obtained here show that higher concentrations of the Group II catalyst significantly impacted the porous character observed in the final gel.

There is little difference in the results obtained for 20% *w/v* and 30% *w/v* at low R/C, with disparities becoming more evident only at higher R/C, especially for the increased Ca loadings. As the xerogels are created within an identical reaction volume but with different masses of solids within that volume, the expectation would be for 20% *w/v* xerogels to demonstrate higher porosities, as less of the volume is occupied by gel; however, this is not observed in all cases. This difference can be ascribed to effects arising from the drying process used. At a low R/C, the gel structures formed are generally stronger than for the higher R/C ratios, where less catalyst is present; this means that the xerogels can more easily withstand the capillary and vacuum forces applied to their structures, and the systems collapse to a similar degree; therefore, the porosity is similar. At a higher R/C, the gel structures are weaker, especially for 20% *w/v*, which fills less of the synthesis volume, and 100% Ca, which is destabilised by the sole presence of the Group II cation; therefore, there is greater collapse and the porosity is reduced. This is validated by surface area trends obtained, as well as the consideration of the combined nitrogen and MIP volumes, as shown in Table 2 and Table 3.

It is evident that the simultaneous addition of Na and Ca produces cluster sizes between those obtained for the individual catalysts, as previously reported [18]. The results obtained here would suggest that similar trends in pore control could be obtained using other combinations; for example, previous work has shown that calcium and barium carbonates create similar final xerogels [18], hence the influence of other Group II species may also result in the moderation of the Group I catalysts. It is also clear that, as the proportion of each cation increases, there is a shift towards the dominant character of the pure gel of that catalyst; however, for the mixed catalyst xerogels, the dominance of Na decreases as the R/C increases, and it is possible to increase the surface areas and pore volumes while maintaining pore size, which has implications for the synthesis of tailored materials and final properties. In these cases, more Ca character develops in the gel; however, it should be noted that there can be issues with solubility for CaCO_3,_ affecting the amount of the catalyst that can be used. It has also been reported that the constantly evolving conditions within the reaction volume may affect catalyst solubility, and it is recognised that increased temperature is unfavourable for solubility [23,24]. For the xerogels studied here, there are limited differences between the 20% *w/v* and 30% *w/v* xerogels, which indicates that solubility is not a strong influence within this particular study. Higher R/C xerogels also exhibit larger pore sizes and volumes, which presents a new avenue for tuning these materials to selected applications, including their use in soil amendments, within fuel cells, or in technologies for CCS [25], air separation [26], water treatment [27], electrochemical processes [28], and hydrogen storage [29], including the use of doped variants for enhanced interaction [30]. 

**Figure 6:**
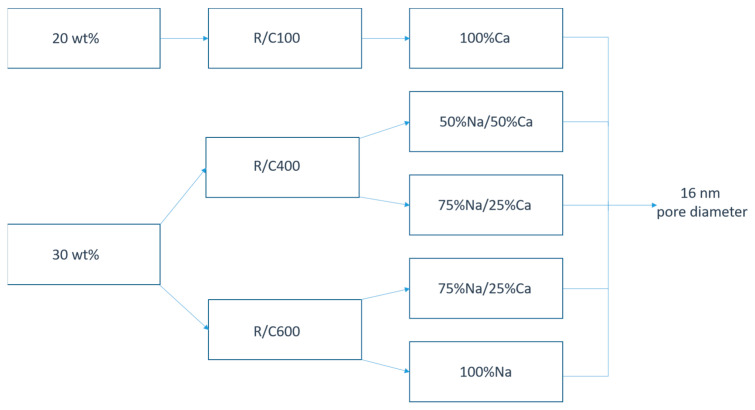
A schematic showing the various routes by which a gel with the same average pore diameter can be obtained using changes in the solids content, R/C ratio, and catalyst ratio.

Figure 6 shows alternative synthesis pathways for obtaining a final gel with an average pore diameter of 16 nm; this can be accomplished through a number of combinations of solids content, catalyst concentration, and catalyst composition. The fact that there are several alternative pathways to the same average pore size identifies many routes to produce controlled properties within the final materials; for example, the systems listed in Figure 6 demonstrate surface areas in the range of 216–426 m^2^ g^−1^, allowing optimisation while retaining average pore size. More notable is the change in monodispersion within the system and the widening of the pore size distribution, providing a route for creating materials with a more selective pore range, which are the very monodispersed materials that we have previously reported [17]. This suggests that the overall properties of RF xerogels can be tailored for the final materials to a high degree with the control of several key variables.

## 3. Conclusions

The synthesis of RF xerogels has been widely investigated; however, the work presented here looks at the tailored synthesis of final xerogels to control their porous character through a combination of catalyst species. The simultaneous addition of Group I and Group II metal carbonates (Na_2_CO_3_ and CaCO_3_) provides a wide contrast of surface areas, pore volumes, and average pore diameters, the latter variable also exhibiting variation in the distribution obtained, which may lead to key advances in the study of materials manipulation. The role of the metal ions is evident in creating feasible xerogels, with Ca resulting in weak, unviable xerogels for some systems; this, combined with the low solubility of Ca, means that the realisation of extremely similar xerogels with reduced Group II carbonate contributions may be favoured for gel synthesis, particularly at scale.

## 4. Materials and Methods

### 4.1. RF Gel Synthesis

Resorcinol (ReagentPlus, 99%), aqueous formaldehyde solution (37 wt% formaldehyde stabilised with 10–15% methanol), Na_2_CO_3_ (anhydrous, ≥99.5%), and CaCO_3_ (≥99%) were used as purchased from Sigma-Aldrich UK.

The RF solution compositions were determined using a metal carbonate mixture, the R/C molar ratio, and the solids content chosen. The total solution volume was fixed at 60 mL, with a resorcinol to formaldehyde (R/F) molar ratio of 0.5, for all gel samples produced. Solids contents of both 20 and 30% weight by volume were studied, resulting in a total mass of R, F, and C of 12 and 18 g, respectively. Depending on the metal carbonate mixture and the R/C ratio chosen, the relative masses of R, F, and C were adjusted accordingly in order to maintain the overall solids content. All preparations were carried out at room temperature.

The RF solution preparation followed a previously established procedure [17,18]. Briefly, the required mass of R was dissolved in 50 mL of deionised water (Millipore Elix 5) in a sealable 500 mL jar with the aid of magnetic stirring. Upon the full dissolution of R, the chosen metal carbonate mixture was added to the solution whilst maintaining magnetic stirring. As CaCO_3_ possesses very limited solubility in water, complete dissolution was not possible when higher concentrations were used. For these cases, additional stirring time (30 min) was added to encourage the maximum dissolution to occur. After the defined period of agitation, the required volume of formaldehyde solution was added to the reaction mixture. This was followed by the addition of more deionised water, i.e., the volume required to produce a final gel volume of 60 mL. Once all components were added to the reaction vessel, the lid was tightly sealed and the solution was allowed to stir for 30 min to ensure thorough mixing and the initiation of the reaction. Subsequently, the magnetic stirrer was removed and the vessel was moved to an oven preheated to 85 ± 5 °C, wherein the vessel remained for three days to allow for gelation and curing. On removal from the oven, the gel was allowed to cool before the exchange of the water within the gel network for acetone. In a slight modification of the established procedure hitherto followed, after the gel was cut into pieces, 100 mL of acetone (ACS reagent, ≥ 99.5%, Sigma-Aldrich) was added to the vessel and the sample was shaken. After 24 h, the used acetone was drained from the system and replaced by 100 mL of fresh acetone, which was, in turn, drained and replaced after a further 24 h. After a third 24 h-exchange period, all the acetone was drained from the gel and the sample was dried under vacuum for two days at 85 °C to produce the finished xerogel product.

Gel nomenclature follows the form of WCa/XNa_Y Z%, where W is the percentage of the catalyst mass contributed by CaCO_3_ and X (100-W) as the remaining contribution from Na_2_CO_3_, Y is the R/C molar ratio, and Z is the solids content. For example, a sample produced with 75% of the catalyst mass from CaCO_3_ and the remaining 25% from Na_2_CO_3_, R/C of 600, and a solids content of 20% would be labelled as 75Ca/25Na_600 20%.

### 4.2. Xerogel Characterisation

Each dried xerogel sample was characterised using nitrogen sorption measurements (Micromeritics ASAP 2420) in order to determine the surface area and porosity. In each case, ~0.25 g of xerogel was accurately weighed into a sample tube and degassed under vacuum at 110 °C for 2 h. This process removed any residual solvent or contaminants from the xerogel surface. Subsequently, low temperature (−196 °C, as maintained by the use of liquid nitrogen) nitrogen sorption analysis was performed, consisting of 40 adsorption points and 30 desorption points.

The Brunauer–Emmett–Teller (BET) theory was applied to each of the nitrogen uptake isotherms [31]. The adsorption branch of each isotherm was analysed between the relative pressures of 0.05 and 0.3, allowing for the determination of the BET surface area (S_BET_) [31]. Pore size distributions and average (mean) pore sizes were calculated by applying the Barrett–Joyner–Halenda (BJH) theory to the desorption branches of the isotherms [32]. While the BJH model is not strictly applicable to the pores generally observed in these RF materials, the prevalence with which the model is used throughout the literature, in addition to the absence of any other practical method of pore size determination, indicated that the BJH theory be applied for this body of work.

Gels that exhibited pore sizes beyond the range detectable by the gas adsorption techniques were further analysed using mercury intrusion porosimetry (MIP) in order to fully quantify the porous volume within the samples. Measurements were conducted using a Quantachrome PoreMaster 60, operating at a low-pressure stage between vacuum and 50 psia (345 kPa), and a high-pressure stage between 20 and 40000 psia (between 137 kPa and 276 MPa). This allowed for the detection of all pores between approximately 5 nm and 250 μm in diameter.

## Figures and Tables

**Table 1:** The catalyst mixtures for dual catalyst resorcinol–formaldehyde xerogels produced in this study.

**% Na_2_CO_3_**	100	75	50	25	0
**% CaCO_3_**	0	25	50	75	100

**Table 2:** The textural properties obtained for resorcinol–formaldehyde xerogels produced using 20% solids content.

%Na	%Ca	S_BET_ (m^2^/g)	PV_N2_ (cm^3^/g)	d_p_ avg. (nm)	PV_Hg_ (cm^3^/g)
R/C 100					
100	0	516 ± 2	0.34	3	-
75	25	556 ± 2	0.41	3	-
50	50	541 ± 1	0.52	4	-
25	75	524 ± 2	0.72	6	-
0	100	396 ± 2	1.18	15	-
R/C 400					
100	0	438 ± 2	1.00	11	-
75	25	413 ± 2	1.01	12	-
50	50	319 ± 2	1.29	19	0.92
25	75	300 ± 1	1.51	28	1.10
0	100	75 ± 1	0.14	11	1.33
R/C 600					
100	0	305 ± 1	1.01	18	0.62
75	25	68 ± 1	0.12	11	0.91
50	50	181 ± 1	0.95	30	1.09

**Table 3:** The textural properties obtained for the resorcinol–formaldehyde xerogels produced using a 30% solids content.

%Na	%Ca	S_BET_ (m^2^/g)	V_N2_ (cm^3^/g)	d_p_ avg. (nm)	V_Hg_ (cm^3^/g)
R/C 100					
100	0	528 ± 2	0.34	3	-
75	25	497 ± 2	0.37	3	-
50	50	554 ± 2	0.54	4	-
25	75	488 ± 2	0.83	8	-
0	100	302 ± 1	1.14	13	-
R/C 400					
100	0	475 ± 2	0.99	10	-
75	25	373 ± 2	1.16	16	-
50	50	426 ± 2	1.26	16	0.79
25	75	290 ± 1	1.65	33	1.51
0	100	304 ± 1	1.30	23	1.55
R/C 600					
100	0	387 ± 1	1.13	16	0.69
75	25	216 ± 1	0.58	16	1.92
50	50	289 ± 1	1.19	25	1.63
25	75	96 ± 1	0.22	14	3.84
0	100	103 ± 1	0.22	13	2.92

## Data Availability

Not applicable.
